# Supplementary material for: Therapeutic effects of skin repair plus ointment on molecular and morphological changes in burn wounds: A rat model
Source: PLoS One. 2025 Dec 19;20(12):e0338305. doi: 10.1371/journal.pone.0338305 (PMC12716683; doi:10.1371/journal.pone.0338305)
Supplement: Attachment — Submitted filename: Dear Academic Editor and Reviewers.docx [file pone.0338305.s002.docx]

Dear Academic Editor and Reviewers,

We would like to sincerely thank you for the valuable time and effort you have devoted to reviewing our manuscript entitled *“Therapeutic Effects of Skin Repair Plus Ointment on Molecular and Morphological Changes in Burn Wounds: A Rat Model”* (PONE-D-25-31940). We deeply appreciate the constructive feedback, which has greatly helped us to improve the quality and clarity of our work.

Below, we provide a point-by-point response to each of the comments raised by the reviewers and the editor.

*** Editorial requirements**: We have carefully addressed all editorial requirements, as detailed below:

**1. PLOS ONE style requirements**
We have revised the manuscript to fully conform with PLOS ONE formatting guidelines, including file naming conventions, as per the provided templates.

**2. Copyediting of the manuscript**
The manuscript has been thoroughly copyedited for grammar, spelling, and clarity by Professor Mustafa Ababneh (Professor of Molecular Virology/ Jordan university of Science and Technology). We believe this has improved the readability and presentation of the manuscript.

**3. Role of funders**
We have amended the funding statement to include the following:
*“The funders had no role in study design, data collection and analysis, decision to publish, or preparation of the manuscript.”*

**4. Data Availability Statement**
We confirm that the submission contains the complete minimal data set required to replicate the study findings. All relevant data, including values used for statistical analyses, graphs, and figures, have been provided within the manuscript and Supporting Information files.

**5. Data sharing plan**
We have ensured that all data will be freely accessible at the time of acceptance, in accordance with PLOS ONE’s open data policy.

The following statement was added in the availability of data and materials section:

The supplementary for the real-time qPCR run files and raw data are available on GitHub at https://github.com/mbalzghoul/Burning-project.

**6. Abstract consistency**
We have amended the abstract so that the version in the manuscript and the version in the online submission form are now identical.

The abstract was corrected to be identical in both manuscript and online submission form.

* Below, we provide a detailed, point-by-point response to each of the reviewers’ comments. **Reviewers’ comments** are presented in **bold**, followed by our responses in *regular text*:

**Reviewer #1**

**1. Statistical power and interpretation: The sample size per time point (n = 3) is too small. Please conduct a power analysis or justify the sample size. Many outcome measures did not reach significance. Acknowledge limitations. Include post-hoc comparisons/effect sizes.**

**Response:**

The following statement was added (Despite our findings indicating substantial increases in TGF-β (on day 3) and IGF-1 (on day 9), other molecular and histopathological parameters did not attain statistical significance. The lack of significance should be interpreted with caution, as it may be partially attributable to the limited per-time point sample size (n = 3). A post hoc power analysis indicated that the present design was sufficiently powered to detect large effect sizes (Cohen’s d ≥ 1.2), but underpowered to detect small-to-moderate differences. Indeed, several outcomes, including VEGF (day 12) and PDGFb (day 3), showed moderate-to-large effect sizes despite not achieving statistical significance, suggesting possible biological relevance. These trends highlight the potential therapeutic effect of Remanco that warrants confirmation in larger, more adequately powered studies. These data demonstrate a substantial early response to Remanco cream, which may be pivotal in expediting the initial stages of wound healing. Transforming growth factor-beta (TGF-β), widely recognized for its role in regulating inflammation and facilitating tissue regeneration, exhibited a notable increase in expression levels during the early stages of the therapy course. On days 9 and 18, TGF-beta expression doubled and tripled, respectively (Fig 4-A). This increase is significant because TGF-beta is essential for orchestrating the deposition of extracellular matrix and the formation of granulation tissue, both of which are critical phases in the healing process [22].)

A limitations section was added to describe all limitations of this study.

This study has several limitations. The small number of animals sacrificed at each time point (n = 3) limited statistical power to detect subtle differences among treatment groups. Our post hoc power analysis confirmed that the study was only adequately powered to detect large effect sizes, so smaller but biologically meaningful effects may have gone undetected. Replication with larger sample sizes is essential to validate the observed trends. The molecular analysis was restricted to a selected panel of growth factors, so including additional markers like pro-inflammatory cytokines, angiogenic regulators, and extracellular matrix components could provide a more comprehensive understanding of Remanco’s effects. Animal models offer valuable mechanistic insights, but translation to human burn wound healing requires further clinical studies.

**2. Gene expression analysis: Non-significant fold changes should be cautiously presented. Consider protein-level validation.**

*Response:*

We thank the reviewer for this important point. We have revised the manuscript to present the non-significant fold changes with explicit caution, framing them as preliminary observations. We fully agree on the necessity of protein-level validation and have acknowledged this as a key limitation and a critical objective for future work to confirm the biological relevance of these transcriptional trends.

**3. Histopathological evaluation: Was scoring blinded?**

Response:

Yes, the histopathological evaluation was performed in a blinded manner

**4. Data presentation: Include summary tables/graphs for wound reduction.**

*Response:* We have added summary graphs to visually depict wound area reduction over time (Figure 1,5).

**5. Discussion and claims: The claim of “cosmetic appearance” and hair regrowth should be supported or removed.**

*Response:* We have removed the unsupported claims of cosmetic improvement and hair regrowth. The Discussion has been reframed to emphasize the preliminary nature of our findings and to present Remanco as a promising traditional formulation requiring further validation.

**6. Minor comments: Clarify figure legends, cite comparative studies with silver sulfadiazine.**

*Response:*

Thank you for these suggestions. We have revised the figure legends to be more comprehensive and have added citations to relevant comparative studies that utilize silver sulfadiazine as a benchmark for burn wound treatment

**Reviewer #2**

**1. Lack of statistical significance in key outcomes. Please add recent literature citations.**

*Response:* We have reframed the Discussion to avoid overstating non-significant outcomes and to highlight the preliminary nature of our results. In addition, we have updated the literature review to include recent publications within the last five years.

**2. Incomplete molecular validation: Only TGF-β and IGF-1 were significant. Provide primer references. Discuss active compounds in Remanco.**

*Response:*

The following table was edited to provide the accession number.

| **Gene** | **Forward Primer (5′→3′)** | **Reverse Primer (5′→3′)** | **Accession number** |
| --- | --- | --- | --- |
| IGF-1 | AAAGTCAGCTCGTTCCATCC | GTTTCCTGCACTTCCTCTACTT | X06043 M17714 |
| PDGF-AA | CACACGCCAGACTGTGTATAA | CATGGTGATGCCTTTGTTTCTC | L41623 |
| PDGF-BB | GAGCCAAGACACCTCAAACT | ATCTCCTTCAGTGCCTTCTTG | PQ117545.1 |
| PDGF-DD | CCATTCGCAGGAAGAGAAGTAT | GCTCCGAGGTATCTCGTAAATG | AB003156.1 |
| TGF-β | CTGAACCAAGGAGACGGAATAC | GTTTGGGACTGATCCCATTGA | M55431.1 |
| Keratinocyte FGF | AGCGACACACGAGAAGTTATG | CCTTTCACTTTGCCTCGTTTG | X56551 |
| FGF7 | AGCGACACACGAGAAGTTATG | CCTTTCACTTTGCCTCGTTTG | NM_002009.4 |
| VEGF | CAATGATGAAGCCCTGGAGT | TCTCCTATGTGCTGGCTTTG | AY378102.1 |
| FGF2 | GACCCACACGTCAAACTACA | GCCGTCCATCTTCCTTCATAG | NM_001077585.1 |
| β-Actin | ACAGGATGCAGAAGGAGATTAC | ACAGTGAGGCCAGGATAGA | EF156276.1 |
| GAPDH | ACTCCCATTCTTCCACCTTTG | CCCTGTTGCTGTAGCCATATT | AF106860.2 |

*Response:* Remanco is a commercially available preparation, and its phytochemical profile is described by the manufacturer. While a full chemical analysis was beyond the scope of this study, we have added available information.

**3. Animal experiment design: Clarify number of animals sacrificed per day. Justify sample collection days. Standardize across figures and tables.**

*Response:*

We thank the reviewer for this critical comment regarding the experimental design. We have now clarified these details in the revised Methods section.

The sampling strategy was as follows: Three animals from each group were randomly selected and sacrificed at each predetermined time point (Days 3, 9, 15, 21, and 30 post-burn) for tissue collection. This resulted in a total sample size of n=3 for histological and molecular analyses at each time point for each group.

The time points were selected to systematically capture key phases of the wound healing process: the early inflammatory phase (Day 3), the proliferative phase (Days 9-15), and the maturation/remodeling phase (Days 21-30). This standardized schedule ensured consistent and comparable sample collection across all groups and for all analyses, including histopathology and gene expression. We have verified that all figures and corresponding legends now explicitly reflect this consistent sample size (n=3 per group per time point)

**4. Missing visual data: Include all figures, graphs, sample sizes, and histological images.**

*Response:*

Thank you for highlighting this. We have now included all missing visual data in the revised manuscript. The submission now contains the complete set of figures and graphs. Furthermore, we have added the corresponding histological images to provide direct visual evidence supporting the scoring and conclusions.

**5. Histopathological data presentation: Please provide representative images.**

*Response:* Representative stained histological sections illustrating re-epithelialization and angiogenesis have been added (Figure 2-4).

**6. Overinterpretation of findings: Reframe conclusions.**

*Response:* We have revised the Discussion and Conclusion to avoid overinterpretation. The study is now framed as preliminary, highlighting the need for larger, more rigorous trials.

**7. Phytochemical characterization: Provide chemical analysis of Remanco.**

*Response:* Remanco is a commercially available preparation, and its phytochemical profile is described by the manufacturer. While a full chemical analysis was beyond the scope of this study, we have added available information.

**8. Minor comments (grammar, study design diagram, graphical abstract).**

*Response:* The manuscript has been thoroughly copyedited for grammar and style.

We are grateful for the reviewers’ constructive critiques, which have helped us substantially improve our manuscript. We have strengthened the statistical analysis, improved data presentation, clarified methods, revised the discussion to avoid overstatement, and improved the overall readability and formatting. We respectfully submit the revised manuscript for your consideration.

Sincerely,
Dr. Foad Alzoughool
On behalf of all co-authors
